# Phenotypic Dimensions of Spirituality: Implications for Mental Health in China, India, and the United States

**DOI:** 10.3389/fpsyg.2016.01600

**Published:** 2016-10-27

**Authors:** Clayton H. McClintock, Elsa Lau, Lisa Miller

**Affiliations:** Department of Clinical Psychology, Teachers College, Columbia UniversityNew York, NY, USA

**Keywords:** spirituality, religion, cross-cultural research, psychopathology, protective factors

## Abstract

While the field of empirical study on religion and spirituality in relation to mental health has rapidly expanded over the past decade, little is known about underlying dimensions of spirituality cross-culturally conceived. We aimed to bridge this gap by inductively deriving potential universal dimensions of spirituality through a large-scale, multi-national data collection, and examining the relationships of these dimensions with common psychiatric conditions. Five-thousand five-hundred and twelve participants from China, India, and the United States completed a two-hour online survey consisting of wide-ranging measures of the lived experience of spirituality, as well as clinical assessments. A series of inductive Exploratory Factor Analysis (EFA) and cross-validating Exploratory Structural Equation Modeling (ESEM) were conducted to derive common underlying dimensions of spirituality. Logistic regression analyses were then conducted with each dimension to predict depression, suicidal ideation, generalized anxiety, and substance-related disorders. Preliminary EFA results were consistently supported by ESEM findings. Analyses of 40 spirituality measures revealed five invariant factors across countries which were interpreted as five dimensions of universal spiritual experience, specifically: love, in the fabric of relationships and as a sacred reality; unifying interconnectedness, as a sense of energetic oneness with other beings in the universe; altruism, as a commitment beyond the self with care and service; contemplative practice, such as meditation, prayer, yoga, or qigong; and religious and spiritual reflection and commitment, as a life well-examined. Love, interconnectedness, and altruism were associated with less risk of psychopathology for all countries. Religious and spiritual reflection and commitment and contemplative practice were associated with less risk in India and the United States but associated with greater risk in China. Education was directly associated with dimensions of spiritual awareness in India and China but inversely associated with dimensions in the United States. Findings support the notion that spirituality is a universal phenomenon with potentially universal dimensions. These aspects of spirituality may each offer protective effects against psychiatric symptoms and disorders and suggest new directions for treatment.

## Introduction

The field of empirical study on religion and spirituality (R/S) in relationship to mental health has rapidly expanded in the past decade. There now exist five peer-reviewed international journals that publish on R/S and its relationship to mental health (*Journal of Religion and Health, Psychology of Religion and Spirituality, Spirituality in Clinical Practice, International Journal of Psychology*, and *Religion and Social Scientific Study of Religion*), and over 4000 articles in this field have been published in the past decade.

Research from this body of work has shown that spirituality and religiosity serve as protective factors against a variety of psychiatric conditions, including for depressive disorders (Koenig et al., 1998; Miller et al., 2012; Barton et al., 2013), anxiety disorders (Azhar et al., 1994; Kaplan et al., 2005), suicide (Dervic et al., 2004; Rasic et al., 2009), and substance-related disorders (Miller and Gur, 2002; Harden, 2010). A systematic and comprehensive review of empirical studies from the past two decades in this field concluded that strong evidence currently exists that religious involvement correlates with better mental health for depression, alcohol and substance abuse, and suicide and that some evidence exists for stress-related disorders and dementia (Bonelli and Koenig, 2013).

### Biological bases of spirituality

Biological markers of the protective benefits of spirituality against depression have been identified and published in high impact peer review journals. Among adults with a sustained personal spirituality over 5 years, MRI findings show cortical thickness in those regions of the brain (parietal, occipital, precuneous) to show cortical thinness in people with recurrent depression (Miller et al., 2014). The findings published in JAMA Psychiatry were interpreted to suggest that personal spirituality may be neuro-protective against depression. In a related study employing EEG, posterior high amplitude alpha was identified in people with a strong personal spirituality who have recovered from depression (Tenke et al., 2013). Greater diurnal regulation of cortisol also was found in people with a strong personal spirituality (Dedert et al., 2004).

Evidence from genetic epidemiologic twin-studies shows that a personal spirituality (either experienced within a religion or outside of a religion) is an innate human faculty, with about 30% of the variance in the strength of personal spirituality among adults attributed to broad heritability (Kendler et al., 1997) and 70% attributed to shared environment and unique environment. Innate spirituality is independent of personality but for a small association with openness to experience (Francis, 1999; Kendler et al., 1999; Piedmont, 1999). Spiritual awareness surges in adolescence with physical puberty, as marked by a 50% in increase in the heritable contribution from middle adolescence to emerging adulthood (Koenig et al., 2005; Button et al., 2011).

### Research challenges

The expression of innate spirituality has yet to be extensively studied in large samples. Moreover, measurement of spirituality in these epidemiologic studies generally has been on single-item measures of importance of spirituality, a lived sense of spiritual relationship with the transcendent, or a sense of the sacred in daily life. These very broad constructs might hold universal dimensions, which if found around the globe, we might consider as phenotypes of innate spirituality. To date, the small handful of large-sample published studies on the protective benefits of R/S have confronted a set of challenges:

1. *Limited assessments of R/S consider only a small number of constructs, typically measured by single items.* Scholars generally agree that religion and spirituality represent complex, multi-dimensional constructs (Emmons, 2000; Hill and Pargament, 2003), yet studies in this field have largely relied on single measures like religious attendance (Strawbridge et al., 2001; Chatters et al., 2008; Reyes-Ortiz et al., 2008), personal importance of spirituality or religion (Miller et al., 2012, 2014), and religious affiliation (Larson and Larson, 1994; Kendler et al., 1997; Dervic et al., 2004) or on a small number of constructs (Cacioppo and Brandon, 2002).
2. Constructs derived from research rely on samples predominantly from North America with nearly exclusively Judeo-Christian affiliations. To the best of our knowledge, there has yet to be published a peer-reviewed article on universal constructs of spirituality, derived from a global sample of countries of diverse religious traditions, as it relates to mental health.
3. *Constructs rely on top-down a priori concepts, rather than inductively derived dimensions of spirituality.* To the best of our knowledge, only one study has inductively examined universal dimensions of spiritual expression, focusing on adolescence during the surge or “biological clock” of spiritual emergence. Benson et al. (2012) in a landmark study on spiritual formation in the second decade collaborated with investigators across 11 countries (*N* = 6725) to showed common processes in spiritual emergence, individuation, and formation in adolescence. For instance, in all countries, as part of spiritual formation, adolescents consider it important to draw their actions in line with their spiritual beliefs and quest for spiritual or transcendent experience. However, as the study focused on global processes of emergence, it did not seek to identify fundamental core spiritual orientations or phenotypes that might persist into adulthood. The investigators also were not focused on a link between universal spirituality and psychopathology.

### Religion and spirituality

In the research literature, scholars generally agree that spirituality and religion represent highly overlapping constructs which refer to human beings' relationship to the transcendent, sacred, and ultimate dimensions of existence (Emmons, 2000). Spirituality is an innate capacity through which we experience transcendence and may extend to lived practices and values. Religion refers more to the beliefs, sacred language and rituals, holy texts, traditions, and institutions which are inextricably contextualized by culture (Geertz, 1973) and that hold and cultivate a natural capacity for spirituality. Among adults, level of personal spirituality and personal adherence to religion correlate about 0.2–0.3 (Kendler et al., 1997; Wang et al., 2003). It would make sense that innate spirituality, whether experienced within or without a religious tradition, might unfold in universal ways.

To contribute toward the ongoing conceptual refinement and global inquiry, the current study administers a very broad range of conceptualization of R/S on a sample of over 5500 participants (20% Buddhist, 21% Christian, 11% Hindu, 2% Muslim, 26% Non-religious, and 9% Other) over three countries with diverse religious demographics, cultural history, governmental relationship to religion and spirituality, and, of course, ethnic diversity. In order to account for individuals who consider themselves spiritual but not religious as well as for the profound diversity between and within particular religious traditions, we sought to measure a construct that includes the very real resources contained within religious traditions while also capturing the natural human capacity for transcendence not necessarily bound by the cultural institutions of religion. This consideration becomes even more important when taking into account multiple religious traditions. For the purposes of this study, therefore, the term *spirituality* signifies personal perceptions, expressions, views, and practices related to a realm of human existence experienced as transcendent and of ultimate concern, irrespective of religious affiliation or belief system (Tillich, 2001; Wilber, 2007; Berry, 2011).

### Aims of the study

The present study asks two primary questions. First, what are basic dimensions, or underlying factors, of a cross-cultural spirituality as assessed in China, India, and the United States? Secondly, how do specific spiritual dimensions individually relate to common psychiatric disorders in these countries?

## Method

### Participants

From June of 2014 to February of 2015, 5512 participants (41% women, mean age = 29.0 years, age range: 18–75 years) were recruited from the crowdsourcing websites Zhubajie.com and Mturk.com among residents of China (*N* = 3150), India (*N* = 863), and the United States (*N* = 1499). An extensive online questionnaire was delivered through the sites, which consisted of a number of widely used spiritual, clinical, and psychological instruments (see *Measures*), as well as sociodemographic questions. For the sample living in China, the original language of English was translated into Chinese. Following guidelines from previous international studies (Squires et al., 2013; WHO, 2015), a committee of bilingual translators translated, back-translated, and pretested the full survey instrument over several iterations to ensure conceptual equivalence across cultures. The institutional review board of Teachers College, Columbia University, approved the study.

### Measures

#### Spirituality measures

We sought to inductively derive a cross-cultural construct of spirituality in a broad and pan-culturally appropriate manner. Little et al. (1999) argued that, for concepts that are broad and not easily defined, maximizing the heterogeneity of indicators optimizes representation of multivariate constructs. To this end, we reviewed the empirical literature on spirituality and religion and collected more than 150 published self-report measures that have direct relevance to personal spiritual experience, whether as perceptions of reality, transcendent or sacred practices, views, or expressions. After several iterations of qualitative analyses, we narrowed down the pool to 54 previously validated measures based on comprehensiveness and appropriateness for a multi-national and multi-religious sample. Forty such measures showed adequate or better reliability in the current sample (alpha coefficients 0.65–0.97) and were included in the analyses.

The Fetzer Multidimensional Measurement of Religiousness/Spirituality assessed overall spirituality, overall religiosity, private religious/spiritual practices, negative religious coping, forgiveness, daily spiritual experiences, overall religious coping, and religious commitment (Fetzer Institute/National Institute on Aging Working Group, 1999). The Intrinsic Religiosity subscale of the Duke University Religion Index assessed intrinsic religiosity and spirituality (Koenig and Büssing, 2010). Salience of spiritual beliefs was measured with the Belief Salience Scale (Blaine and Crocker, 1995). Mystical experience was measured with the Mysticism Scale (Hood, 1975). Compassion was assessed by the Compassion subscale of the Dispositional Positive Emotions Scale (Shiota et al., 2006). Sitting and moving contemplative practice frequency were each assessed by average number of sessions per month and number of total months of practice. Two items assessed the presence and importance of spiritual role models, and seven items were used to measure spirituality in nature. Positive morality was measured by the Positive Morality subscale of the Prague Spirituality Questionnaire (Rican and Janosova, 2010). Spiritual quest was measured by the Quest scale (Batson and Ventis, 1982). Religious meaning was measured with the Religious Meaning Scale (Krause, 2003). Experiences of ontological, psychological, social, and religious love were assessed by respective subscales within the Sorkin Multidimensional Index of Love Experience (Levin, 2000). Spiritual self-discovery, spiritual relations, sense of sacredness, and eco-awareness were assessed by respective dimensions within the Spirituality Scale (Delaney, 2005). The Universality, Prayer Fulfillment, and Connectedness subscales of the Spiritual Transcendence Scale assessed a sense of unity in life, contentment with prayer, and connection to others, respectively (Piedmont, 1999). The Spiritual Transcendence Index was a measure of spiritual transcendence (Seidlitz et al., 2002). Humanistic engagement, religious engagement, existential engagement, spiritual study and practice, and gratitude and awe were assessed by subscales of the SpREUK-P Questionnaire (Büssing et al., 2005). Self-transcendence was assessed with the Self-Transcendence subscale of the Temperament and Character Inventory (Cloninger, 1999).

#### Psychopathology measures

Psychiatric symptoms were assessed with the Patient Health Questionnaire (PHQ-9; Kroenke et al., 2001), the General Anxiety Disorder scale (GAD-7; Spitzer et al., 2006), and Monitoring the Future (Johnston, 2010). Cut-off scores were then used to dichotomize the variables as clinically significant and clinically non-significant levels. Based on previous studies (Löwe et al., 2008; Manea et al., 2012), major depression was defined as a score of 10 or greater on the PHQ-9; suicidal ideation was defined as a score of one or greater on question nine of the PHQ-9; and generalized anxiety was defined as a score of 10 or greater on the GAD-7. In addition, alcohol-related disorder was defined as being “drunk or very high from alcohol” six or more times in the past 30 days. Similarly, cannabis-related disorder was defined as using cannabis six or more times in the past 30 days.

### Statistical analyses

The raw data were analyzed using SPSS 22.0 (SPSS I, 2011) and Mplus 7.0 (Muthén and Muthén, 2012). To thoroughly examine, replicate, and cross-validate the factor structure, the total sample was first randomly divided into four sub-samples. Contemplation variables were treated as censored variables (Muthén and Muthén, 2012). Using oblique quartimin rotation and a weighted least square means and variance adjusted (WLSRV) estimator, the first and second samples were used to perform an exploratory factor analyses (EFA) using all of the original items. Acceptability of the factor models was evaluated by goodness of model fit, interpretability of the solution, and strength of parameter estimates (Brown, 2015).

Based on the EFAs, the third and fourth samples were used to perform exploratory structural equation modeling (ESEM) with WLSRV estimation and quartimin rotation to validate the factor structure of spirituality. The ESEM approach differs from the typical confirmatory factor analysis (CFA) approach in that all factor loadings are estimated (Asparouhov and Muthén, 2009; Marsh et al., 2009). While CFA is a commonly used method that has certain advantages over EFA methods, statisticians have recently argued that CFA models usually lead to distorted factors with overestimated factor correlations and typically do not provide even a minimal standard of fit to the data (Marsh, 2007; Marsh et al., 2010, 2005; Schmitt and Sass, 2011). Even when CFA does provides an acceptable fit, ESEM has been shown not only to provide a better fit but also to derive latent factors that are much more differentiated (Marsh et al., 2009). The ESEM model fit was examined using the Tucker-Lewis Index (TLI), comparative fit index (CFI), and root mean square error of approximation (RMSEA), as operationalized in Mplus in association with the WLSMV estimator (Muthén and Muthén, 2012). We also used a multi-group ESEM model to test configural invariance across countries (Marsh et al., 2013). Guidelines for acceptable fit have typically been interpreted for the TLI and CFI as >0.95 and 0.90 for good and acceptable fit, and for the RMSEA as < 0.05 and 0.08 as good and acceptable fit (Marsh et al., 2004). However, it is important to note that statistical researchers have cautioned against a strict application of such guidelines, as there is considerable evidence that realistically large factor structures are typically unable to satisfy even minimal acceptable standards of fit (Beauducel and Wittmann, 2005; Marsh et al., 2005). Standardized residuals and modification indices (MIs) were used to determine the presence of any localized areas of strain in the solutions (Brown, 2015).

After EFA and ESEM analyses, we conducted remaining analyses on the full sample (*N* = 5512). To simplify interpretation of odds ratios, and because previous research demonstrated the protective effects of a high level of spirituality (Miller et al., 1997, 2012), each dimension was first dichotomized into the top quartile for each country (high) and all others (low). To determine whether each of the spirituality factors individually predicted the occurrence of psychopathology, a univariate logistic regression of each dimension was conducted with each measure of psychopathology as the dichotomous outcome variable. Age, gender, and educational levels were included in all analyses to control for potential confounds. Spearman's correlation with these sociodemographic variables was also conducted with each dimension.

## Results

### Religious characteristics

**Figure 1:**
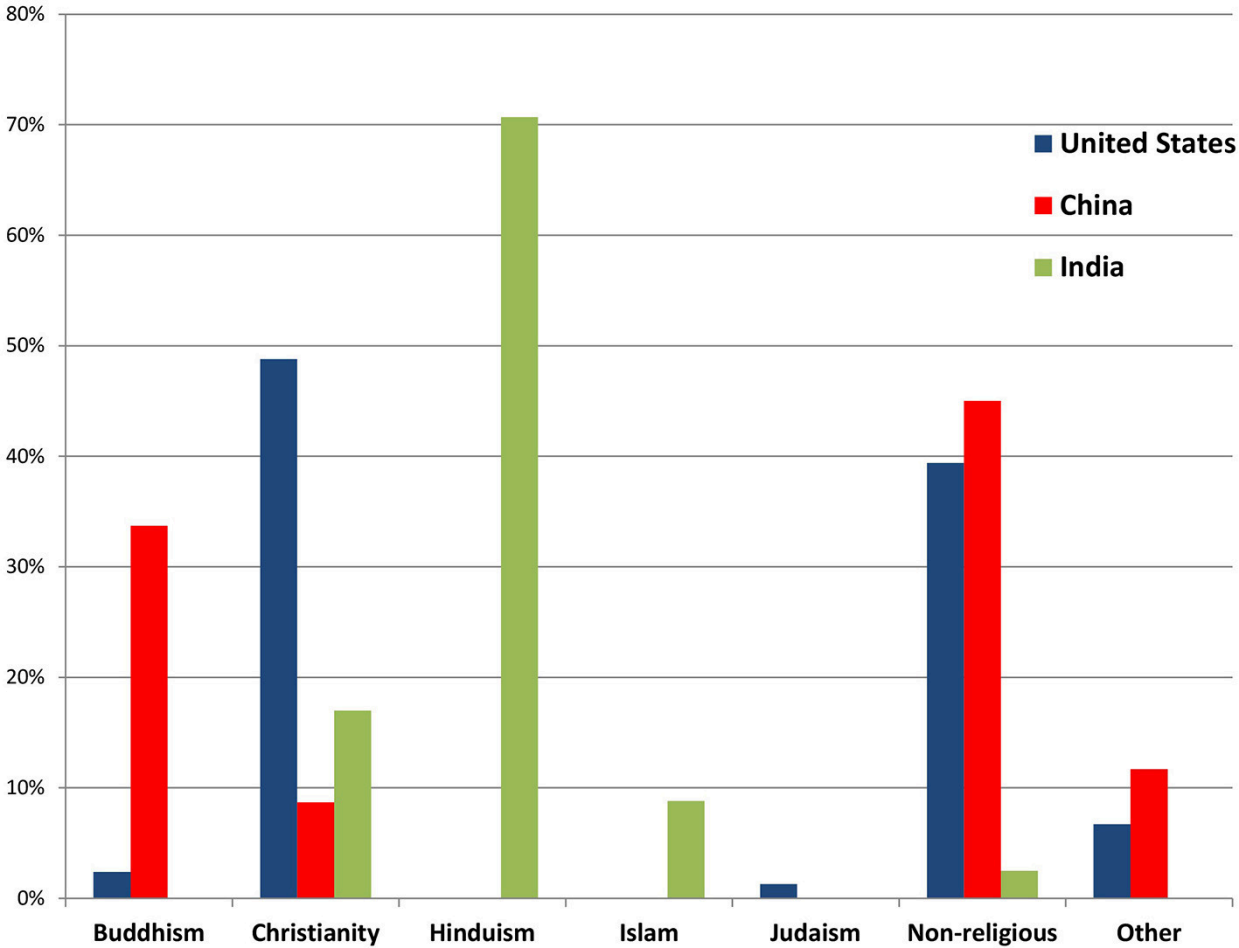
**Religious affiliation of community samples in China, India, and the United States (*N* = 5512)**.

As Figure 1 shows, a plurality (45.0%) of the Chinese sample identified as non-religious, while a third (33.7%) were Buddhist. In the Indian sample, the vast majority (70.7%) identified as Hindu, while Christians were the second most populous group (17.0%). In the United States sample, the predominant religious affiliation was Christianity (48.8%) followed closely by non-religiously affiliated (39.4%) (see also Supplementary Table 5).

### EFA

An EFA in sample 1 (*N* = 919) produced six eigenvalues exceeding unity. The five-factor EFA model was considerably more interpretable than the six-factor solution. To maximize the clarity of the model, we retained only those measures that showed strong primary loadings without salient cross-loadings. After six measures that had salient cross-loadings were removed, an EFA with the remaining 34 items produced five eigenvalues >1, with all items clearly loading onto one factor without salient cross-loadings (Table 1).

**Table 1:** **Latent structure of 34 spirituality measures: exploratory factor analyses in samples 1 and 2 and exploratory structural equation modeling in samples 3 and 4**.

**Dimension**																				
	**Religious and spiritual reflection and commitment**				**Contemplative practice**				**Unifying interconnectedness**				**Love**				**Altruism**			
**Measure**	**S1**	**S2**	**S3**	**S4**	**S1**	**S2**	**S3**	**S4**	**S1**	**S2**	**S3**	**S4**	**S1**	**S2**	**S3**	**S4**	**S1**	**S2**	**S3**	**S4**
Belief salience	**0.87**	**0.91**	**0.88**	**0.89**	0.01	−0.01	0.02	0.02	0.09	−0.01	0.07	0.06	0.03	0.03	0.03	0.01	−0.08	−0.04	−0.07	−0.07
Overall religiosity	**0.90**	**0.89**	**0.88**	**0.84**	−0.02	0.02	0.02	0.05	−0.11	−0.12	−0.11	−0.12	0.02	−0.03	0.04	0.09	−0.04	0.03	−0.05	−0.06
Intrinsic religiosity/spirituality	**0.85**	**0.87**	**0.87**	**0.88**	−0.01	−0.02	−0.03	−0.03	0.05	0.02	0.03	0.03	0.03	0.02	0.01	−0.01	0.01	0.03	0.03	0.04
Religious engagement	**0.87**	**0.85**	**0.87**	**0.86**	0.09	0.12	0.06	0.04	−0.18	−0.17	−0.19	−0.21	−0.04	0.02	−0.01	0.00	0.07	0.05	0.07	0.09
Negative religious coping	**0.86**	**0.85**	**0.85**	**0.86**	0.02	0.03	0.01	0.01	−0.05	−0.03	−0.04	−0.04	0.04	0.00	0.01	−0.02	0.01	0.04	0.03	0.05
Daily spiritual experiences	**0.80**	**0.79**	**0.81**	**0.83**	0.04	0.01	0.02	0.02	−0.07	0.04	−0.05	−0.05	0.02	−0.04	0.01	0.00	0.14	0.14	0.12	0.12
Spiritual transcendence	**0.78**	**0.83**	**0.79**	**0.82**	0.08	0.03	0.06	0.02	0.22	0.12	0.22	0.20	0.03	0.09	0.05	0.06	−0.07	−0.05	−0.06	−0.06
Religious meaning	**0.78**	**0.79**	**0.77**	**0.80**	−0.05	0.01	−0.03	−0.04	0.18	0.12	0.18	0.16	0.02	0.11	0.06	0.08	0.02	−0.06	0.00	−0.01
Religious commitment	**0.78**	**0.84**	**0.77**	**0.77**	−0.05	−0.04	−0.03	0.00	0.03	0.01	0.00	0.02	0.01	−0.03	0.02	−0.01	0.03	−0.02	0.01	0.00
Overall religious coping	**0.79**	**0.77**	**0.78**	**0.75**	−0.01	−0.04	0.01	0.04	0.05	0.10	0.04	0.03	−0.03	−0.10	−0.04	−0.03	0.02	0.05	0.05	0.06
Religious love	**0.76**	**0.77**	**0.76**	**0.77**	−0.07	−0.03	−0.07	−0.09	0.12	0.03	0.10	0.11	0.17	0.24	0.20	0.19	−0.06	−0.09	−0.06	−0.05
Intrinsic spirituality	**0.73**	**0.76**	**0.72**	**0.72**	0.05	0.01	0.06	0.08	0.17	0.18	0.19	0.17	−0.05	−0.06	−0.05	−0.04	0.07	0.01	0.07	0.06
Forgiveness	**0.71**	**0.76**	**0.71**	**0.74**	−0.08	−0.03	−0.10	−0.08	0.04	0.06	0.06	0.06	0.03	0.03	0.00	−0.02	0.16	0.04	0.16	0.13
Private religious/spiritual practice	**0.74**	**0.66**	**0.74**	**0.70**	0.17	0.20	0.18	0.22	−0.19	−0.12	−0.23	−0.26	−0.04	−0.05	−0.04	0.02	0.00	0.06	0.00	−0.01
Spiritual role model	**0.57**	**0.52**	**0.57**	**0.57**	0.07	0.11	0.09	0.10	0.00	0.01	0.01	0.03	0.12	0.09	0.06	−0.01	0.00	−0.03	0.01	0.00
Overall spirituality	**0.53**	**0.61**	**0.53**	**0.55**	0.16	0.03	0.12	0.11	0.14	0.20	0.19	0.19	−0.08	−0.13	−0.08	−0.06	0.17	0.15	0.17	0.16
Moving contemplation frequency	−0.08	−0.06	−0.10	−0.10	**0.76**	**0.89**	**0.91**	**0.92**	0.00	−0.01	0.00	0.02	0.03	0.06	0.05	0.03	−0.04	−0.05	−0.03	−0.05
Moving contemplation lifetime	−0.09	−0.06	−0.10	−0.10	**0.75**	**0.86**	**0.89**	**0.90**	0.01	0.01	0.00	0.03	0.03	0.03	0.04	0.02	−0.03	0.00	−0.01	−0.01
Sitting contemplation frequency	0.08	0.08	0.19	0.17	**0.80**	**0.83**	**0.75**	**0.80**	0.01	0.02	0.01	−0.04	0.02	−0.04	−0.03	−0.02	0.03	0.02	0.05	0.04
Sitting contemplation lifetime	0.08	0.08	0.18	0.16	**0.77**	**0.79**	**0.72**	**0.77**	0.02	0.05	0.02	−0.02	0.00	−0.07	−0.05	−0.03	0.05	0.06	0.07	0.07
Spirituality in nature	0.13		0.15	0.19	0.20		0.17	0.17	**0.53**		**0.51**	**0.55**	0.06		0.13	0.14	0.07		0.07	0.04
Spiritual eco-awareness	−0.03	−0.04	0.00	0.00	0.14	0.12	0.10	0.12	**0.53**	**0.64**	**0.48**	**0.48**	0.22	0.19	0.27	0.29	0.15	0.17	0.19	0.21
Unity in life	0.27	0.24	**0.31**	**0.35**	0.05	0.08	0.04	0.03	**0.55**	**0.66**	**0.48**	**0.45**	0.07	−0.02	0.10	0.08	0.03	−0.07	0.03	0.06
Positive morality	0.28		0.26	0.27	0.01		0.02	0.06	**0.45**		**0.42**	**0.41**	0.05		0.14	0.18	0.09		0.07	0.04
Spiritual quest	0.06		0.04	0.12	0.10		0.10	0.07	**0.40**		**0.41**	**0.40**	0.00		0.02	0.01	0.01		0.05	0.04
Spiritual self-discovery	0.17	0.20	0.21	0.18	0.20	0.14	0.18	0.22	**0.44**	**0.55**	**0.39**	**0.38**	0.19	0.10	0.23	0.26	0.07	0.11	0.09	0.10
Connection to others	0.10	0.04	0.16	0.16	0.00	0.05	0.02	0.01	**0.41**	**0.48**	0.26	0.25	0.22	0.10	0.24	0.19	0.10	0.11	0.12	0.17
Psychological love	0.03	0.04	0.04	0.05	0.03	0.01	0.02	0.01	−0.05	0.00	−0.05	−0.04	**0.87**	**0.84**	**0.88**	**0.87**	−0.08	−0.02	−0.07	−0.09
Social love	0.01	−0.03	−0.04	−0.07	−0.03	−0.01	0.00	0.00	−0.04	0.00	−0.04	−0.03	**0.74**	**0.65**	**0.76**	**0.78**	0.19	0.29	0.20	0.19
Ontological love	0.05	0.13	0.09	0.11	0.07	0.02	0.04	0.01	0.13	0.12	0.12	0.11	**0.70**	**0.68**	**0.72**	**0.70**	0.06	0.06	0.05	0.08
Humanistic engagement	−0.01	0.06	0.00	−0.01	−0.03	0.03	−0.03	−0.03	−0.07	−0.03	−0.11	−0.10	0.06	0.06	0.07	0.08	**0.92**	**0.88**	**0.92**	**0.92**
Gratitude and awe	0.06	0.12	0.05	0.07	0.05	0.11	0.06	0.06	0.08	0.16	0.10	0.12	0.03	0.06	0.04	0.03	**0.75**	**0.55**	**0.70**	**0.68**
Existential engagement	0.14		0.14	0.20	0.23		0.16	0.14	0.21		0.27	0.23	−0.06		−0.05	−0.06	**0.53**		**0.58**	**0.59**
Compassion for others	−0.09	−0.12	−0.10	−0.13	−0.11	−0.13	−0.03	0.04	0.20	0.27	0.15	0.12	0.21	0.15	0.23	0.28	**0.38**	**0.47**	**0.40**	**0.37**

S1, Sample 1 (N = 919); S2, Sample 2 (N = 919); S3, Sample 3 (N = 1837); S4, Sample 4 (N = (1837). Factor loadings ≥ |0.30| are in boldface. EFA and ESEM were conducted with with WLSRV estimation and quartimin rotation. A priori target loadings for ESEM models are shaded in gray.

A second EFA was conducted with sample 2 (*N* = 919). Five factors had eigenvalues >1. Ten measures had salient cross-loadings and, thus, were removed. An EFA with the remaining 30 measures again produced five eigenvalues exceeding one. The same five factors emerged and all items loaded onto the same factors that they did in the first EFA (Table 1).

The measures for mystical experience, contentment with prayer, sense of sacredness, spiritual relationships, self-transcendence, and spiritual study and practice were observed to cross-load in both EFAs, suggesting they were not clearly related to a single latent factor. Thus, an EFA was rerun on the combined samples 1 and 2 (*N* = 1838) after removal of these six items. Again, five eigenvalues exceeded unity, primary factor loadings were identical to the previous EFAs, and no salient cross-loadings were present. Thus, we excluded the six items and retained the rest for subsequent analyses.

### ESEM

ESEM was applied to sample 3 (*N* = 1837) to cross-validate the EFA solutions involving the retained items. The initial five-factor model yielded indices below levels of acceptable fit (RMSEA = 0.056, CFI = 0.822, TLI = 0.750). However, this model indicated localized areas of strain (i.e. MIs). Since the areas of strain were substantively justified (Brown, 2015), the model was re-specified to allow the following residuals to freely co-vary as correlated uniquenesses (CU): connection to others with unity of life; eco-awareness with spiritual self-discovery; existential engagement with compassion for others; spiritual quest with spirituality in nature; and connection to others with compassion for others. The revised model provided marginally acceptable fit to the data (RMSEA = 0.041, CFI = 0.908, TLI = 0.870). A multi-group ESEM model that included these CUs was applied and provided a similar fit to the data (RMSEA = 0.043, CFI = 0.899, TLI = 0.857).

Because the CUs were designated afterwards based on MIs, the ESEM model with sample 3 was considered exploratory (Brown, 2015), and a second ESEM was conducted on sample 4 (*N* = 1837) in which the CUs from the previous model were applied a priori. This model, similarly, provided marginally acceptable fit to the data (RMSEA = 0.044, CFI = 0.890, TLI = 0.845), as did the multi-group model (RMSEA = 0.043, CFI = 0.897, TLI = 0.854). Table 1 presents the factor loadings obtained in the ESEMs. All 34 measures except for connection to others had salient primary loadings in both samples, and no measure except for unity in life showed a salient cross-loading.

### Factor structure

Of the five factors, the first factor, labeled “religious and spiritual reflection and commitment,” displayed primary loadings on items that measure a commitment to religious and spiritual beliefs, communities, and practices and the integration of these into one's life. Measures of belief salience, religious engagement, and intrinsic religiosity and spirituality loaded highly onto this dimension. The second factor, labeled “contemplative practice,” loaded onto the frequency of contemplative practices, like meditation, contemplative prayer, qigong, and yoga. This factor included measures for frequency of participation in meditative and mind-body practices.

The third factor, called “unifying interconnectedness,” displayed loadings primarily on ways in which one consciously perceives a connection to other people and forms of life. Measures of spirituality in nature, eco-awareness, positive morality, and unity in life loaded highly on this factor. The fourth factor, labeled “love,” loaded onto items that reflect feelings of love for others and oneself. This dimension included measures of psychological, ontological, and social love. The fifth factor, “altruism,” demonstrated primary loadings on items that indicate noticing and helping others. Measures of humanistic engagement, gratitude and awe, and compassion for others were included in this dimension.

Interfactor correlations between the five dimensions, weighted by country, ranged from +0.28 to +0.56 (Table 2). The religious and spiritual reflection and commitment factor highly correlated with the most commonly used single-item spirituality/religiosity measures of importance of religiosity and spirituality (+0.79), religious service attendance (+0.75), and religious affiliation (+0.68), correlations which were much higher than with the other dimensions (see also Supplementary Table 6).

**Table 2:** **Inter-factor correlations for the five spirituality dimensions and three commonly used single-item spirituality measures**.

	**Religious and spiritual reflection and commitment**	**Contemplative practice**	**Unifying interconnected-ness**	**Love**	**Altruism**	**Importance of religiosity or spirituality**	**Religious service attendance**	**Religious affiliation**
Religious and spiritual reflection and commitment	1.00							
Contemplative practice	0.55	1.00						
Unifying interconnectedness	0.55	0.36	1.00					
Love	0.48	0.28	0.52	1.00				
Altruism	0.50	0.36	0.37	0.56	1.00			
Importance of religiosity or spirituality	0.79	0.42	0.40	0.36	0.40	1.00		
Religious service attendance	0.75	0.48	0.23	0.35	0.38	0.62	1.00	
Religious affiliation	0.68	0.38	0.34	0.32	0.27	0.56	0.57	1.00

All correlations are significant at the p < 0.01 level.

### Spiritual dimensions and sociodemographics

Table 3 lists correlations between spiritual dimensions and sociodemographic variables in each country. Most of the dimensions were positively associated with age in each country. Females also were more likely than males to have a high level of spirituality in each country. Education had a positive association between dimensions of spirituality in China and India; however, in the United States, education was inversely associated with love and unifying interconnectedness.

**Table 3:** **Correlations between spirituality dimensions and sociodemographic variables among community samples in China, India, and the United States**.

**Characteristic by country**	**Dimension**				
	**Religious and spiritual reflection and commitment**	**Contemplative practice**	**Unifying interconnectedness**	**Love**	**Altruism**
**CHINA**					
Age	0.09^**^	0.06^**^	0.06^**^	0.06^**^	0.01
Gender^a^	−0.01	−0.01	0.11^**^	0.09^**^	0.07^**^
Education^b^	0.06^**^	0.02	0.07^**^	0.06^**^	0.07^**^
**INDIA**					
Age	0.07^**^	0.09^**^	−0.01	0.04	0.05^*^
Gender^a^	0.07^**^	0.06^*^	−0.01	0.02	0.02
Education^b^	0.04	0.10^**^	0.01	0.04	0.01
**UNITED STATES**					
Age	0.14^**^	0.09^**^	0.07^**^	−0.01	0.13^**^
Gender^a^	0.15^**^	0.03	0.08^**^	0.06^*^	0.12^**^
Education^b^	0.02	0.03	−0.05^*^	−0.06^*^	−0.01

a *Positive values indicate association in women; negative values indicate association in men*.

b *Units measured by the following rank order categories: high school degree or some high school; associates degree or some undergraduate; undergraduate degree; graduate degree*.

* *p < 0.05*,

** *p < 0.01*.

### Spiritual dimensions and psychopathology

Table 4 lists for each of the five spirituality factors the odds ratios of major depression, suicidal ideation, generalized anxiety disorder, alcohol-related disorder, and cannabis-related disorder, with age, gender, and education level controlled (see also Supplementary Table 7). Participants who had high religious and spiritual reflection and commitment had less risk for virtually all psychiatric conditions in India and the United States but a greater risk in China. Similarly, high contemplative practice was associated with greater risk in China for all conditions but in India was related to decreased risk for most conditions. Follow-up analyses revealed that gender moderated the direct correlations between these two factors and psychopathology in China, such that these results held true for men but not for women. In all countries, high awareness of unifying interconnectedness was inversely correlated with risk of depression and suicidal ideation; furthermore, in China it was inversely related to substance-related disorders. Participants with high experience of love and altruism showed decreased odds of having virtually any condition in all three countries.

**Table 4:** **Odds ratios of psychiatric disorders and suicidal ideation in China, India, and the United States associated with dimensions of spirituality**.

**Spiritual dimension by country**	**Condition^a^**									
	**Major depressive disorder**		**Suicidal ideation**		**Generalized anxiety disorder**		**Alcohol-related disorder**		**Cannabis-related disorder**	
	**OR**	**95% CI**	**OR**	**95% CI**	**OR**	**95% CI**	**OR**	**95% CI**	**OR**	**95% CI**
**CHINA (*****N*** = **3150)**										
**Spirituality dimensions**										
Reflection and commitment	1.31^**^	1.10–1.55	1.38^**^	1.16–1.63	1.55^**^	1.27–1.89	2.53^**^	1.86–3.45	3.37^**^	2.27–5.01
Contemplative practice	1.06	0.89–1.26	1.07	0.90–1.27	0.98	0.80–1.21	2.18^**^	1.60–2.98	2.19^**^	1.46–3.27
Unifying Interconnectedness	0.81^*^	0.67–0.96	0.56^**^	0.47–0.67	0.78^*^	0.63–0.97	0.42^**^	0.26–0.66	0.16^**^	0.07–0.40
Love	0.81^*^	0.67–0.96	0.65^**^	0.54–0.77	0.77^*^	0.62–0.96	0.48^**^	0.31–0.75	0.20^**^	0.09–0.45
Altruism	0.63^**^	0.53–0.76	0.53^**^	0.44–0.64	0.70^**^	0.56–0.87	0.51^**^	0.33–0.78	0.40^**^	0.22–0.73
**INDIA (*****N*** = **863)**										
**Spirituality dimensions**										
Reflection and commitment	0.45^**^	0.32–0.64	0.60^**^	0.43–0.84	0.48^**^	0.33–0.71	0.31^*^	0.12–0.78	0.51	0.22–1.16
Contemplative practice	0.62^**^	0.44–0.87	0.68^*^	0.48–0.95	0.67^*^	0.46–0.97	0.73	0.36–1.49	1.06	0.54–2.10
Unifying Interconnectedness	0.50^**^	0.36–0.71	0.55^**^	0.40–0.77	0.85	0.59–1.21	0.99	0.53–1.84	1.18	0.62–2.23
Love	0.40^**^	0.28–0.56	0.41^**^	0.29–0.57	0.38^**^	0.26–0.57	0.46^*^	0.21–0.99	0.44^*^	0.19–1.00
Altruism	0.28^**^	0.19–0.40	0.38^**^	0.27–0.53	0.28^**^	0.18–0.43	0.21^**^	0.08–0.59	0.31^*^	0.12–0.79
**UNITED STATES (*****N*** = **1499)**										
**Spirituality dimensions**										
Reflection and commitment	0.44^**^	0.32–0.61	0.54^**^	0.38–0.78	0.60^**^	0.42–0.85	0.28^**^	0.12–0.65	0.54^*^	0.31–0.94
Contemplative practice	0.83	0.62–1.11	1.01	0.74–1.38	0.95	0.69–1.30	0.68	0.38–1.21	1.06	0.68–1.67
Unifying Interconnectedness	0.64^**^	0.48–0.87	0.62^**^	0.44–0.88	0.82	0.59–1.14	0.65	0.36–1.19	1.08	0.69–1.68
Love	0.42^**^	0.30–0.57	0.53^**^	0.37–0.75	0.67^*^	0.48–0.93	0.72	0.41–1.27	0.89	0.56–1.40
Altruism	0.50^**^	0.36–0.68	0.48^**^	0.33–0.70	0.69^*^	0.49–0.97	0.53^*^	0.27–1.01	0.83	0.51–1.34

a *In these univariate models, the outcome measures are the dichotomous variables representing the presence or absence of the listed psychiatric conditions, and the predictors are dichotomous variables representing high vs. low of each spirituality dimension in each country. Age, gender, and education are controlled*.

* *p < 0.05*,

** *p < 0.01*.

## Discussion

This study sought to identify global, cross-cultural dimensions of spirituality in India, China, and the United States. Five inductively derived dimensions of spirituality were found across the three countries: love, in the fabric of relationships and as a sacred reality; unifying interconnectedness, as a sense of energetic oneness with other beings in the universe; altruism, as a commitment beyond the self with care and service; a contemplative practice, such as meditation, prayer, yoga, or qigong; and religious and spiritual reflection and commitment, as a life well-examined. We view these findings as an initial step which will next need replication in a broad range of countries representing diverse religious and cultural traditions.

The study further found a relationship between these five spiritual dimensions and risk of internalizing psychopathology in China, India, and the United States. Greater awareness of love, interconnectedness, and altruism were universally associated with decreased levels of depression, suicidal ideation, anxiety, and substance-related disorders. Religious and spiritual reflection and commitment and contemplative practice similarly were inversely associated with a range of clinical disorders in India and the United States but directly associated with disorders in China. In assessing the impact of demographics, we found that a greater level of education was associated with a higher level of spirituality along the five dimensions in India and China; however, in the United States, inverse associations were found between education and the spiritual dimensions of love and unifying interconnectedness.

### Dimensions of spirituality across cultures

Given both the diverse pool of relevant measures and the diverse countries represented in the sample, the dimensions that emerged empirically bestow a rich and differentiated cross-cultural portrait of spirituality. While we do not claim complete coverage of all possible facets of human spirituality, five distinct and relevant factors emerged. The relative lack of distinction between measures that conceptually tap either religious or spiritual constructs within underlying factors is consistent with previous factor analyses and demonstrates that the domains of spirituality and religion have significant conceptual overlap and, in some ways, may be indistinguishable (Kendler et al., 2003; Benson et al., 2012).

These dimensions of spirituality were found to relate to the assessed sociodemographic factors within each country. With the notable exception of inverse relationships with education in the United States, age, female gender, and level of education were found to directly correlate with each of the dimensions. These findings are generally consistent with previous research on sociodemographic factors of spirituality (Chatters et al., 1992; Levin et al., 1994; Assari, 2015). Older age has predicted higher levels of spirituality and religiosity in numerous studies, suggesting that on average spiritual views and practices intensify with age (Moberg, 1971; Levin, 1989; Levin and Taylor, 1993). Previous investigations also show that women consistently are more spiritually and religiously inclined, though explanations for this phenomenon vary widely and have little consensus (Argyle and Beit-Hallahmi, 1975; Taylor, 2002; Sprecher and Fehr, 2005; Bryant, 2007). The relationship with education and other measures of socioeconomic status is much more variable. Some studies have found direct correlations, which may suggest a social integration of religious and spiritual values and practices (Mueller, 1980; Taylor, 1988; Cornwall, 1989). Others have found inverse correlations, which has been interpreted as evidence that spirituality and religion provide resources for dealing with socially and materially disadvantaged life circumstances (Stark, 1972; Koenig et al., 1988; Paul, 2010; Piff et al., 2010). The inverse correlations in the current study are discussed further in Section Spirituality and Education.

Importantly, the statistical invariance of the dimensions across countries—remarkable in itself given the religious diversity of the sample—strongly suggests that experiences of human spirituality are universal across national and religious cultures (Greenwald and Harder, 2003; Wilson, 2012; Murdock, 1945). The cross-cultural and multi-dimensional structure of spirituality that emerged does not imply that real differences in the particularities of both traditional and non-traditional spiritual expressions and experiences do not exist; rather, it provides a skeletal framework for understanding important components of a potentially universal spirituality.

A strong degree of convergence emerged with previous factor analytical studies of spirituality and religiosity. Religious and spiritual reflection and commitment is conceptually very similar to Kendler et al. (1997, 2003) personal devotion and general religiosity, and McDonald's (MacDonald, 2000; MacDonald et al., 2015) religiousness and cognitive orientation toward spirituality factors. Furthermore, it highly correlated (+0.68 to +0.79) in the present study with the measures of religiosity most commonly used in the field: personal importance of spirituality/religion, religious service attendance, and religious affiliation (Bonelli and Koenig, 2013). Contemplative practice has significant overlap with Benson et al. (2012) spiritual and religious practices factors, as well as the constructs of mindfulness (Kabat-Zinn, 2003) and the relaxation response (Benson, 1982) as they are applied in clinical settings, both of which trace their origins to spiritual traditions (Cobb et al., 2016). Unifying interconnectedness has much in common with McDonald's (MacDonald, 2000) experiential/phenomenological dimension and Greenwald and Harder's (2003) blissful transcendence. The experience of love has significant overlap with Kendler et al. (2003) forgiveness factor and Greenwald and Harder's (2003) loving connection to others. Finally, altruism is very similar to Greenwald and Harder's (2003) self-effacing altruism as well as Benson et al. (2012) connecting with others through prosocial beliefs and actions and spirituality in action factors. The striking similarities to other factor analytic studies, despite considerable differences in instruments and samples, lend additional support to the validity of the dimensions of spirituality that emerged in this study.

### Relationship between spiritual dimensions and psychopathology

Beyond adding to our understanding of spirituality *per se*, these findings have important clinical implications as well. While the spiritual dimensions were found for the most part to be associated with lower risk for major depression, generalized anxiety, suicidal ideation, and substance-related disorders, the strongest and most consistent inversely related factors in all three countries were altruism and love. Across countries, those in the top quartile of altruistic engagement compared to all others had a 37–72% reduction in odds of having major depressive disorder, 47–62% reduction for suicidal ideation, 30–72% reduction for generalized anxiety disorder, and 47–79% reduction for alcohol abuse. Those in the top quartile of experience of love showed 19–60% less likelihood of having major depressive disorder, 35–49% decrease for suicidal ideation, and 23–62% decrease for generalized anxiety disorder.

This set of findings represents a significant contribution to the literature given that previous studies in this area have almost entirely used measures of religious participation and personal importance of religion and spirituality (Larson et al., 1978; Miller et al., 2012; Bonelli and Koenig, 2013). In the current study, altruism and love were observed to have higher inverse correlations for nearly all conditions in all three countries compared to these single-item measures. This result clearly illustrates the advantages of measuring the complex construct of spirituality in a multi-dimensional and multi-variate fashion (Cacioppo and Brandon, 2002).

Though we cannot make inferences on the temporal direction of the associations based on the cross-sectional design of this study, previous studies on altruism, compassion, and prosocial behavior strongly suggest that these characteristics have a causal influence on clinical symptoms. Studies indicate that focusing attention away from oneself decreases ruminative and self-referential processing in the default network, associated with depression and unhappiness (Berman et al., 2010; Brewer et al., 2011; Hamilton et al., 2011). Weng et al. (2013) found that training in compassion enhanced neural mechanisms that support fronto-parietal executive control, reward processing, and understanding of other people's emotional states. Furthermore, altruistic responses in this study were correlated with the same changes in neural systems. The very capacity to give support to others in need has also been found to activate self-compassion (Breines and Chen, 2013), which itself has been found to robustly reduce clinical symptoms (Van Dam et al., 2011; MacBeth and Gumley, 2012). In other words, having compassion for and serving others draws upon the same inner resources needed to help oneself through difficult experiences.

Also consistent with our findings, love and similar positively valenced emotions have been shown in previous studies to accord lasting dispositional effects that support mental health and resilience (Fredrickson et al., 2008; Garland et al., 2010). The experience of love has been found to activate the brain's reward system (Panksepp et al., 1994), to increase oxytocin and vasopressin receptors (Keverne and Kendrick, 1992), to reduce HPA axis activity (Carter, 1998), and to deactivate brain regions associated with negative emotions (Zeki, 2007), all of which confer health benefits and decrease clinical symptoms (Carter, 1998). Research has also found that deficits in loving attachments and feelings of belongingness are risk factors for psychopathology (Baumeister and Leary, 1995). Though most of these studies were conducted within the context of family relationships, these findings suggest that an ongoing relational bond with a higher power and a sense of belongingness in the universe accord similar protective effects. Vaillant (2008) has posited a fundamental role within spiritual experience for positive social emotions like love, which “free the self from the self” (p. 50).

In all three countries, an awareness of unifying interconnectedness with other beings was related to less risk for major depression and suicidal ideation. Notably, feeling connected to the natural world makes up a significant aspect of this dimension. Experiments have shown that exposure and a sense of connection to one's natural surroundings improves mood and decreases rumination (Berman et al., 2008; Mayer et al., 2008; Bratman et al., 2015a). In addition, Bratman et al. (2015b) found that experiencing a natural setting reduced neural activity in the subgenual prefrontal cortex, which is linked to behavioral withdrawal and rumination. Studies that examine social networks and social connectivity also support the notion that positive social connections lead to better mental health outcomes (Cohen, 2004; Christakis and Fowler, 2009). Taken together, these findings suggest that an experientially felt sense of connection to others, both human and other species, reduces ruminative thought, elevates overall mood, and decreases risk of psychopathology.

Contemplative practice in India was also found to inversely correlate with psychiatric conditions. Previous research on contemplative practices has found strong evidence that at least certain forms of meditation, most prominently mindfulness meditation, reduces rumination, anxiety, and other forms of psychological distress. A review of 56 studies on mindfulness meditation concluded that mindfulness reduces emotional reactivity and a variety of clinical symptoms and improves overall mental health (Keng et al., 2011). Mechanisms by which meditative practices affect symptomatology have been studied from different angles. There is evidence from mindfulness-based cognitive therapy trials that mindfulness reduces recurrence of depression by decreasing cognitive reactivity (Kuyken et al., 2010). Contemplative training has also been shown to increase aspects of interoceptive awareness (Bornemann et al., 2015), which has been shown to decrease depressive symptoms (Farb et al., 2010). Brewer et al. (2011) and Braam et al. (1997) also found that the default network, associated with unhappiness, was relatively deactivated among experienced meditators compared to non-meditators. Though the vast majority of this research to date focuses on mindfulness meditation, data from these studies strongly suggest that various forms of contemplative practice can lead to a reduction of risk for internalizing psychiatric conditions.

Religious and spiritual reflection and commitment also had a consistent protective effect in India and the United States. In these countries, those in the top quartile of religious and spiritual commitment compared to all others had approximately half the likelihood of having major depressive disorder, suicidal ideation, and generalized anxiety disorder. Conceptually, this factor has the highest overlap with the most commonly used single measures of spirituality—importance of spirituality and religiosity, religious service attendance, and religious affiliation—a notion supported by high bivariate correlations in this study (see Table 3). These findings are consistent with data from studies that use these measures and which have found protective effects against depressive disorders (Braam et al., 1997; Koenig et al., 1998; Miller et al., 2012), suicidality (Hovey, 1999; Cook et al., 2002; Dervic et al., 2004), and anxiety disorders (Azhar et al., 1994). Miller et al. (2014) found that importance of religion or spirituality directly correlated with cortical thickness in regions in the occipital and parietal lobes; moreover, the effect of religious and spiritual importance was stronger for people who had a higher risk of depression in the same neural regions implicated previously in risk for depression. This result suggests that spiritual commitment may confer resilience in part by expanding cortical reserves in regions where cortical thinning poses risk for depressive illness.

Arguably the most complex of the derived spiritual dimensions, religious and spiritual reflection and commitment involves an orientation of one's lived life toward a transcendent power. Whether or not in the context of an established religious tradition, such a commitment inexorably bestows upon an individual a sense of meaning beyond one's own life (Cloninger, 2006), which itself reduces risk of psychopathology and is particularly important during difficult life circumstances (Debats, 1996; Koenig, 2009). Religious and spiritual worldviews also tend to be fundamentally hopeful and have a range of resources for confronting hardship and suffering (Koenig, 2009). Moreover, those who participate in religious and spiritual communities often receive positive support from their community, regardless of economic or social resource (Koenig, 2012).

### Dimensions associated with greater risk in china

A notable exception to the protective effects of spirituality was found in China, as religious and spiritual reflection and commitment and contemplative practice in this country were associated with increased rather than decreased risks of internalizing disorders and symptoms. Interestingly, this discrepant finding is consistent with data on other published studies on spirituality and mental health in China. Wang et al. (2015) observed that importance of religion and religious service attendance in a community population in China directly correlated with incidence of psychopathology, particularly anxiety disorders. In addition, Zhang and colleagues (Zhang and Xu, 2007; Zhang et al., 2011) found that, among Chinese rural women, those who had suicidal intentions and committed suicide were more likely to be religiously affiliated.

Perhaps the differential findings may be associated with differential national policy around religious freedom of expression. Though research in China in this area is still sparse, these findings suggest that the religious climate of the larger society over decades may play an important moderating role. At times, some governmental policies in China have dissuaded religious practitioners from various traditions (Grim and Finke, 2007), which may have contributed to the correlation between higher commitment and contemplation and greater levels of psychopathology. Indeed, the two phenotypes found to be depressogenic are the most overtly religious of the five. In short, not to develop these natural phenotypes can be depressing and demoralizing.

In addition, China, in contrast to India and the United States, has a much greater percentage of non-religious residents, who comprise the majority of the country (Grim and Finke, 2007). Those who are committed to religious beliefs and practices remain outside mainstream culture as minorities may, therefore, likely experience greater internal conflict of values and social alienation (Zhang et al., 2011). This minority status may disproportionately affect men (Zhao et al., 2012). Moreover, China's Confucian-based culture places emphasis on social ties, “saving face,” and individual subordination for the sake of social harmony. Considering all these factors together, psychological strain among religious believers in China may outweigh protective benefits of these particular spiritual phenotypes.

### Spirituality and education

Findings are consistent with research showing lower levels of spirituality among affluent youth in a nationally representative sample of the United States (Barkin et al., 2015). Moreover, other studies have shown elevated rates of substance abuse, depression, and anxiety within this sociodemographic group (Song et al., 2009; Luthar and Barkin, 2012; Patrick et al., 2012). Reasons for this relationship may include an observed overemphasis of outward status and wealth in United States communities high in socioeconomic status (SES), where a sense of self-worth is often tied to external measures of success (Miller, 1995; Luthar, 2003).

Indeed, materialistic cultural values have been on the rise over the past several decades in the United States (Twenge, 2006; Twenge et al., 2012). In tandem with the increase in objective material wealth, research has found that among the college-educated attitudes toward acquiring material wealth has also changed. Between the early 1970's and the late 1990's, first-year students who believed that a “very important” reason to go to college was “to make more money” rose from 50 to 75%. More strikingly, those who considered it “very important or essential” to become “financially very well off” jumped from 39 to 74% during the same time period (Sax et al., 1998; Astin et al., 1987).

Particularly within communities of high SES, pressure to achieve in educational and professional spheres is often enormous, and the competition can be intense, exacting a toll on the interior growth of individuals in such an environment (Chase, 2008). When people individualistically focus on maximizing their own goals, they can feel less connected to people around them (Myers and Diener, 1995). Among upper-middle class children, sports as leisure and spontaneous play has largely been replaced by regimented competition as early as second grade (Luthar et al., 2013). One study found that possession of high income reduced people's ability to savor the pleasures of everyday life (Quoidbach et al., 2010). Overall, evidence points to a culture of privilege within more highly educated circles in the United States that overvalues extrinsic goals and undervalues the intrinsic, a phenomenon which current evidence suggests may not extend to China or India.

### Limitations

This is the first study to examine multiple dimensions of spirituality and their relations to psychopathology in an international sample. As an initial study, our sample size while large is drawn from three diverse countries. Future research will involve data collection in additional countries to include Brazil and Iran. The cross-sectional design does not allow for causal inferences and, therefore, spirituality may alter the risk of clinical symptoms, the symptoms may affect the level of spirituality, or a third factor may influence both. The large sample size captures a wide cross-section of the population yet is limited to people with access to the internet. Nevertheless, large-scale internet-based samples that draw on crowdsourcing have been shown in studies have shown to generate samples more representative of the population than in-person convenience samples (Berinsky et al., 2012; Paolacci and Chandler, 2014). In terms of measures, the method of data acquisition relied on self-report instruments rather than behavioral ratings, or clinician-administered interviews. The instruments we employed, however, have shown high validity and high concordance with clinician administered DSM-IV-TR diagnoses in previous studies (Löwe et al., 2008; Manea et al., 2012). Overall, this study represents the first large-scale study to investigate the possibility of global dimensions of spirituality that may potentially represent phenotypes of innate spirituality.

### Clinical implications

Taken together with previous reports, our finding that dimensions of spirituality are inversely associated with internalizing psychiatric conditions of depression, anxiety, and substance-related disorders offers direction for clinical interventions. Aspects of spirituality, including dimensions found in the present study, have already been integrated and applied in a broad range of empirically-validated treatments for depressive and anxiety disorders (Sperry and Shafranske, 2005; Aten et al., 2014). For instance, contemplative practice and awareness of interconnectedness is fostered in mindfulness-based cognitive therapy (Segal et al., 2012), and along with love, in spiritual awareness psychotherapy (Miller, 2007). Reflection and commitment (an examined life) is part of religiously integrated cognitive behavioral therapy (Pearce et al., 2015), as well as in spiritually oriented therapy (Sperry and Shafranske, 2005). Loving-kindness meditation (Hofmann et al., 2011; Gilbert, 2014) draws upon awareness and cultivation of love and altruism. Indeed, awareness of each of the five spiritual dimensions could have a potentially additive effect together on treatment. With deep respect for the client's religious tradition, in tandem these universal spiritual dimensions can be integrated into most treatments for depression, anxiety, and substance use.

Since, as this study shows, a person's spiritual life has direct relevance to mental health, it follows that a greater curiosity and awareness of a patient's spiritual orientation and practice on the part of mental health practitioners, regardless of therapeutic orientation, would yield clinically useful information. Indeed, formal spiritual assessments have been developed specifically for this purpose (Hall and Edwards, 2002).

## Conclusion

The current study represents an initial investigation into potentially global and universal dimensions of spirituality, which may represent phenotypes of innate spirituality. Five potential phenotypes were identified to include: love, in the fabric of relationships and as a sacred reality; unifying interconnectivity, as a sense of energetic oneness with other beings in the universe; altruism, a commitment beyond the self with care and service; a contemplative practice, such as meditation, prayer, yoga, or qigong; and religious and spiritual reflection and commitment, as a life well-examined. We draw our sample from India, China, and the United states to show a diverse range of religious and cultural expressions, as well as differences in the outward expression of lived religion and spirituality. That universal spirituality exists across these three countries suggests that exploration of universal spirituality in other countries is merited and may offer stronger or clarifying evidence. These findings preliminarily support the notion that spirituality is a universal phenomenon with cross-culturally common dimensions, each of which may offer protective effects against psychiatric symptoms and disorders, and suggest new directions for clinical interventions.

## Author contributions

CM and EL collected the data. CM performed the data analyses and wrote the manuscript. LM supervised the overall project and edited the manuscript.

### Conflict of interest statement

The authors declare that the research was conducted in the absence of any commercial or financial relationships that could be construed as a potential conflict of interest.
