# Supplementary material for: Phenotypic Dimensions of Spirituality: Implications for Mental Health in China, India, and the United States
Source: Front Psychol. 2016 Oct 27;7:1600. doi: 10.3389/fpsyg.2016.01600 (PMC5082226; doi:10.3389/fpsyg.2016.01600)
Supplement: Supplementary file 1 [file Table5.PDF]

Table 5. *Sociodemographic Characteristics of Community Samples in China, India, and the United States (N = 5,512)*

Characteristic	Country						Overall Sample	
	China (*N* = 3150)		India (*N* = 863)		United States (*N* = 1499)			
	Mean	SD	Mean	SD	Mean	SD	Mean	SD
Age	25.2	5.0	31.8	9.3	34.9	10.9	29.0	9.0
	N	%	N	%	N	%	N	%
Gender								
Female	1175	37.4	328	38.0	771	55.4	2274	41.3
Male	1968	62.6	535	62.0	728	48.6	3231	58.7
Education level								
Graduate degree	173	5.5	302	36.5	146	10.0	621	11.4
Undergraduate degree	2011	64.1	420	50.7	666	45.5	3097	57.0
Some undergraduate	611	19.5	74	8.9	433	29.6	1118	20.3
High school degree or some high school	342	10.9	31	3.8	220	15.0	593	10.9
Religious affiliation								
Buddhism	1053	33.7	1	< 1.0	36	2.4	1090	19.9
Christianity	272	8.7	145	17.0	727	48.8	1144	20.9
Hinduism	4	< 1.0	602	70.7	7	< 1.0	613	11.2
Islam	15	< 1.0	75	8.8	13	< 1.0	103	1.9
Judaism	11	< 1.0	1	< 1.0	20	1.3	32	< 1.0
Other	366	11.7	6	< 1.0	100	6.7	472	8.6
Non-religious	1406	45.0	21	2.5	587	39.4	1406	25.7
