# Supplementary material for: Phenotypic Dimensions of Spirituality: Implications for Mental Health in China, India, and the United States
Source: Front Psychol. 2016 Oct 27;7:1600. doi: 10.3389/fpsyg.2016.01600 (PMC5082226; doi:10.3389/fpsyg.2016.01600)
Supplement: Supplementary file 2 [file Table6.PDF]

Table 6. *Inter-factor correlations for the five spirituality dimensions and commonly used spirituality measures by country*

	Religious and Spiritual Reflection and Commitment	Contemplative Practice	Unifying Interconnected-ness	Love	Altruism	Importance of Religiosity or Spirituality	Religious Service Attendance	Religious Affiliation
China (*N* = 3150)								
Reflection and Commitment	1.00							
Contemplative Practice	0.73	1.00						
Unifying Interconnectedness	0.44	0.20	1.00					
Love	0.35	0.16	0.55	1.00				
Altruism	0.38	0.33	0.47	0.57	1.00			
Importance of Religiosity or Spirituality	0.54	0.39	0.27	0.22	0.27	1.00		
Religious Service Attendance	0.60	0.47	0.13	0.17	0.20	0.33	1.00	
Religious Affiliation	0.54	0.40	0.14	0.17	0.13	0.34	0.44	1.00
India (*N* = 863)								
Reflection and Commitment	1.00							
Contemplative Practice	0.63	1.00						
Unifying Interconnectedness	0.38	0.16	1.00					
Love	0.34	0.17	0.53	1.00				
Altruism	0.46	0.38	0.31	0.57	1.00			
Importance of Religiosity or Spirituality	0.62	0.34	0.16	0.14	0.26	1.00		

Religious Service Attendance	0.58	0.42	0.07	0.17	0.24	0.38	1.00	
Religious Affiliation	0.44	0.23	0.21	0.11	0.09	0.28	0.32	1.00
United States (N = 1499)								
Reflection and Commitment	1.00							
Contemplative Practice	0.55	1.00						
Unifying Interconnectedness	0.67	0.60	1.00					
Love	0.38	0.21	0.49	1.00				
Altruism	0.42	0.43	0.52	0.54	1.00			
Importance of Religiosity or Spirituality	0.88	0.46	0.54	0.29	0.34	1.00		
Religious Service Attendance	0.72	0.39	0.32	0.26	0.25	0.64	1.00	
Religious Affiliation	0.74	0.35	0.49	0.28	0.23	0.67	0.59	1.00

All correlations are significant at the $p < .01$ level.
