# Supplementary material for: Phenotypic Dimensions of Spirituality: Implications for Mental Health in China, India, and the United States
Source: Front Psychol. 2016 Oct 27;7:1600. doi: 10.3389/fpsyg.2016.01600 (PMC5082226; doi:10.3389/fpsyg.2016.01600)
Supplement: Supplementary file 3 [file Table7.PDF]

Table 7. *Associations Between Psychiatric Disorders and Suicidal Ideation in China, India, and the United States and Dimensions of Spirituality*

Spiritual Dimension by Country	Condition				
	Major Depressive Disorder	Suicidal Ideation	Generalized Anxiety Disorder	Alcohol-Related Disorder	Cannabis-Related Disorder
China (*N* = 3150)					
Spirituality dimensions					
Reflection and commitment	+	+	+	+	+
Contemplative practice	0	0	0	+	+
Unifying Interconnectedness	-	-	-	-	-
Love	-	-	-	-	-
Altruism	-	-	-	-	-
India (*N* = 863)					
Spirituality dimensions					
Reflection and commitment	-	-	-	-	0
Contemplative practice	-	-	-	0	0
Unifying Interconnectedness	-	-	0	0	0
Love	-	-	-	-	-
Altruism	-	-	-	-	-
United States (*N* = 1499)					
Spirituality dimensions					
Reflection and commitment	-	-	-	-	-
Contemplative practice	0	0	0	0	0
Unifying Interconnectedness	-	-	0	0	0
Love	-	-	-	0	0
Altruism	-	-	-	-	0
